# Microstructure and Properties of Poly(ethylene glycol)-Segmented Polyurethane Antifouling Coatings after Immersion in Seawater

**DOI:** 10.3390/polym13040573

**Published:** 2021-02-14

**Authors:** Kejiao Li, Yuhong Qi, Yingju Zhou, Xiaoyu Sun, Zhanping Zhang

**Affiliations:** Department of Materials Science and Engineering, Dalian Maritime University, Dalian 116026, China; lkj19941128@dlmu.edu.cn (K.L.); zyj180316@163.com (Y.Z.); sxy13188364515@163.com (X.S.); zzp@dlmu.edu.cn (Z.Z.)

**Keywords:** poly(ethylene glycol)-segmented polyurethane coating, seawater immersion, microstructure, properties

## Abstract

Polyurethane has a microphase separation structure, while polyethylene glycol (PEG) can form a hydrated layer to resist protein adsorption. In this paper, PEG was introduced to polyurethane to improve the antifouling properties of the polyurethane, providing a new method and idea for the preparation of new antifouling polyurethane materials. The mechanical properties, hydrophilicity, swelling degree, microphase separation and antifouling performance of the coatings were evaluated. The response characteristics of the polyurethane coatings in a seawater environment were studied, and the performance changes of coatings in seawater were tested. The results showed that the crystallized PEG soft segments increased, promoting microphase separation. The stress at 100% and the elasticity modulus of the polyurethane material also markedly increased, in addition to increases in the swelling degree in seawater, the water contact angle decreased. A total of 25% of PEG incorporated into a soft segment can markedly improve the antibacterial properties of the coatings, but adding more PEG has little significant effect. After immersion in seawater, the coatings became softer and more elastic. This is because water molecules formed hydrogen bonding with the amino NH, which resulted in a weakening effect being exerted on the carbonyl C=O hydrogen bonding and ether oxygen group crystallization.

## 1. Introduction

The ocean is a complex ecological environment. The attachment of marine fouling organisms can cause significant economic losses for ships and underwater facilities [1]. With the increase in human activity and the development of the marine industry economy, the problem of biological attachment has become a worldwide topic.

**Figure 1:**
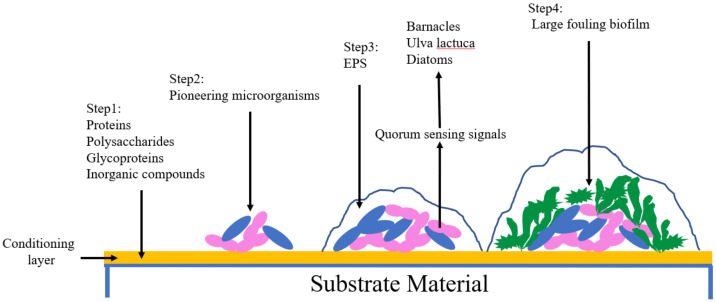
Primary steps in fouling organism’s attachment. EPS: extracellular polymeric substances.

There are several key stages in the attachment of fouling organisms (Figure 1). When hydrophilic materials enter the seawater to form a wet surface, small organic molecules such as protein, polysaccharides, glycoproteins and some inorganic compounds are absorbed via van der Waal’s forces, hydrogen bonding and electrostatic interactions resulting in the formation of a conditioning layer [2]. Subsequently, pioneering microorganisms adhere to the conditioning layer through non-covalent bond forces, forming a biofilm. Because nutrition is provided by running water [3], microorganisms continue to grow, reproduce and expand. Extracellular polymeric substances are secreted (EPS). EPS are not only capable of producing a great enough binding force between the biofilm and the material [4], but can also cover algal spores and protozoa. At the same time, microorganisms send out quorum sensing signals [5,6], attracting crustacea, ulvophyceae and diatoms, which secrete a mucus composed of proteins and polysaccharides which enables the organism to fix itself to the substrate [7], eventually forming a large fouling biofilm.

After exploration, the antifouling effect of antifouling coatings on surfaces was determined to be the best. Due to the increasing demand for environmental protection measures, research directions have gradually changed towards the production of non-toxic environmentally friendly antifouling coatings. The copper-based polymer coating is the most widely studied and used self-polishing antifouling coating. When the Cu^2+^ reaches a certain concentration, it inactivates the main enzymes that marine life depends on. At the same time, protein in biological cells also flocculates, resulting in metal protein sediment, eventually leading to the alteration and death of the biological tissue [8]. Copper-based polymer coatings are generally divided into two types. The first of these types is a soluble acrylic polymer. In water, the side chain of the polymer is hydrolyzed and Cu^2+^ is exchanged with rich metal ions such as Na^+^, K^+^, Ca^2+^ that are present in the seawater. This results in the release of Cu^2+^ and achievement of antifouling performance. The second type of polymer coating refers to insoluble hydrophobic polymers. Seawater enters into the coating through the pores of the coating, leading to release of the antifouling agent, which then diffuses into the seawater eliciting the antifouling effect. As the antifouling agent gradually decreases, the porosity gradually increases, which makes the antifouling agent diffuse more and more easily into the seawater [1]. However, no matter what kind of self-polishing antifouling coating is used, the hydrolysis rate is uncontrollable and the service life is short [9]. Moreover, Cu^2+^ still has a certain level of toxicity which endangers the ecological environment [4].

Low surface energy antifouling coatings include silicone and organic fluorine coating. These rely on low surface free energy to make it difficult for fouling organisms to adhere to the coating. Even if they were to attach, they cannot achieve a firm attachment. When the ship reaches a certain speed, the flowing water provided a certain shear force which resulted in the fouling organisms that were not firmly attached being washed away [10]. On the contrary, if there is no particularly good bonding coating, the low surface energy characteristic makes it difficult to combine the coating with the substrate. For ships that are berthed in the port for a long time or run at a low speed, it is difficult to lose the fouling organisms. Biomimetic antifouling coatings have been designed by bionic technology. Natural active antifouling agents have been extracted from plants and bacteria to replace toxic antifouling agents, such as capsaicin, onions [11] and active substances from marine bacteria [12]. However, the yield of this method is low and sources are limited. The stability of active antifouling agents is poor and the technology is not mature. As a result, there are difficulties associated with the widespread application of this technology. The surface of some animals and plants is not easy to attach to other organisms, such as the microstructure of shark skin [13,14], the fiber structure of dolphin skin [15] and the superhydrophobic surface of the surface of the lotus leaf [16,17]. Through research and imitation of these surface structures, biomimetic coatings have been constructed to achieve the antifouling effect. However, a disadvantage was that a large area of the original structure was not easy to obtain, and the structure was only effective for a single fouling organism. In view of this, antifouling coatings with microphase separation structures have been developed and investigated. The source of biofouling adhesion is the formation of a conditioning layer composed of protein and other substances. However, the microphase separation polymer has the function of inhibiting protein adhesion. When two or more monomers with different properties are polymerized to form block or graft polymers, phase separation tends to occur due to the incompatibility of the thermodynamics and kinetics between the monomers. However, chemical bonds are also present between the monomers, which limits the formation of phase separation on the macro scale. Therefore, phase separation can only be formed at the nanometer or micron scale. The structure formed by different phase regions is called a microphase separation structure [18,19]. The size of the microphase separation structure is usually smaller than the size of protein, causing difficulties in the adhesion of the protein to the surface [20].

**Figure 2:**
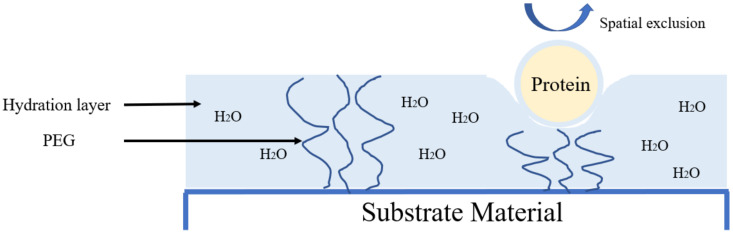
Anti-protein mechanism of polyethylene glycol (PEG).

In addition to microphase separation polymers, other materials possessing their own unique properties are also able to resist protein adsorption. Based on the summary of a large number of experimental data, Ostuni [21] proposed that surface functional groups that resist protein adsorption generally have the following characteristics: (1) they are hydrophilic; (2) they have oxygen bond receptors; (3) they have a non-oxygen bond donor; (4) they are electrically neutral. The most important is the interaction between the surface functional groups and water molecules. Based on this principle, polyethylene glycol (PEG) is the most widely used anti-protein material. PEG is a material with a high hydrophilicity. It is composed of repeated -CH_2_CH_2_O- units. Due to unique structure, it is capable of forming hydrogen bonds with water molecules. At the same time, these hydrogen bonds combine with a large number of water molecules, finally resulting in the forming a hydrated layer on the surface of the PEG material. In order to maintain a high-level structure, protein molecules need water molecules to participate in the formation of necessary hydrogen bonds, and their own surface also has a hydrated layer [22]. So, it is not feasible for proteins to adhere to the surface of PEG materials. In addition, because PEG forms a hydrated polymer chain in water and has a relatively large excluded volume, the molecular chain is compressed and the conformation is limited when the protein approaches. From the point of view of energy, there is an unstable state with reduced entropy [23], so the PEG chain produces a repulsion effect to avoid the protein approaching (Figure 2). 

At present, anti-protein adsorption materials are mainly used in medicine [24,25] and rarely in marine antifouling [26,27,28]. Francolini et al. [25] synthesized poly(ethylene glycol)-grafted segmented polyurethane and characterized it. The results showed that the initial adhesion of bacteria on the surface of the PEG-functionalized polyurethane was essentially inhibited and biofilm formation was also strongly reduced. Isabel et al. [26] developed a hydrophilic polycarbonate-poly(ethylene glycol) methyl ether (mPEG) polyurethane coating. The coating showed a low protein adhesion value and could self-replenish hydrophilicity after being damaged. Holberg et al. [27] synthesized a hydrophilic and biocide-free fouling-release coating by dispersing a polydimethyl siloxane (silicone, PDMS)-polyethylene glycol (PEG) copolymer in a PDMS coating. Compared with steel, the synthesized silicone coating comprising PEG could reduce fouling growth and adhesion. Gu et al. [28] first proposed an acryloxy-terminated polydimethylsiloxane/polyethylene glycol (PEG) blend coating. The study showed that shewanella adhesion is efficiently discouraged by the introduction of PEG segments.

The microphase separation antifouling coatings were affected by their own structures and had the advantages of not creating any pollution and having strong designability. In this paper, PU was used as the main film-forming material and was synthesized by a two-step polymerization reaction. PEG was introduced into the soft segment of PU to form poly(ethylene glycol)-segmented polyurethane (PEG-PU). The introduction of PEG not only promoted microphase separation, but also demonstrated good hydrophilicity and anti-protein ability, which enhanced the antifouling ability of the coating. However, if a large amount of PEG was added, the strong hydrophilicity of PEG would make the coating not adhere to the substrate firmly during the immersion process, and consequently, the degree of microphase separation is greatly reduced. Some researchers have combined low surface energy polysiloxane with polyurethane to improve performance, but the compatibility between the two is poor. This paper attempted to combine hydrophobic PEG and hydrophilic PPG as a soft segment to enhance the basic performance, while ensuring the production of a microphase separation structure and antifouling ability. By comparing the changes in structures, microphase separation degree and antibacterial adhesion of the coating before and after seawater immersion, the influence of PEG contents on the coating was studied, thus determining the optimal proportion of PEG.

## 2. Materials and Methods

### 2.1. Materials

Isophorone diisocyanate (IPDI), polyethylene glycol (PEG, M_n_ = 2000), and trimethylolpropane (TMP) were purchased from Shanghai Aladdin Biochemical Technology Co., Ltd., (Shanghai, China). Compounds 1,4-butanediol (BDO) and polyoxypropylene glycol (PPG, M_n_ = 2000) were purchased from Sinopharm Chemical Reagent Co., Ltd., (Beijing, China). Dibutyltin dilaurate (DBTDL) was purchased from Tianjin Kemiou Chemical Reagent Co., Ltd., (Tianjin, China). PPG and PEG were dewatered at 120 °C under vacuum conditions for 3 h before use. BDO and mixing solvent (dimethylbenzene:isobutylacetate:cyclohexanone = 2:2:1) were dried over a 4A molecular sieve before use. IPDI and DBTDL were used as received.

### 2.2. Preparation of Poly(ethylene glycol)-Segmented Polyurethane Films and Coatings

**Figure 3:**
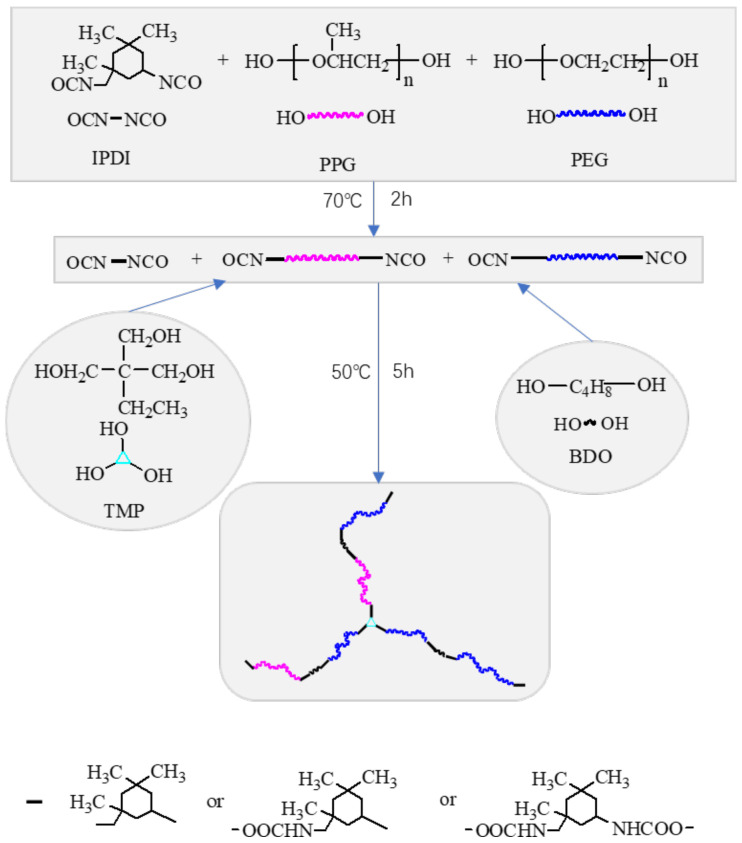
Synthetic route of the Poly(ethylene glycol)-segmented polyurethane (PEG-PU).

Poly(ethylene glycol)-segmented polyurethane (PEG-PU) was synthesized by following a two-step polymerization procedure in a four-neck, round bottom, Pyrex reaction flask equipped with a mechanical stirrer, reflux condenser, addition funnel and nitrogen inlet. In the first step, the calculated amount of PPG, PEG and TDI were introduced into the reactor and slowly heated to 70 °C for 2 h, then cooled to 40 °C. A stoichiometric amount of chain extender BDO and crosslinking agent TMP, dissolved in a mixing solvent and 0.06% of catalyst DBTDL were introduced into the addition funnel and slowly added into the reactor. This was then slowly heated to 50 °C for 5 h to promote the reaction. Figure 3 shows the synthetic route of PEG-PU. The NCO to OH group ratio was 1.3, while the hard segment content (TDI+BDO+TMP) was 38 wt.%, the TMP content was 1.6% (mole). The soft segment content (PPG+PEG) was 62 wt.%. The code PEG50 represents the sample with PEG of 50 wt.% in the soft segment. 

The polymer films with thicknesses of 1–2 mm were obtained by pouring the polymer solution into Teflon molds and coatings with thicknesses of 30–50 μm were obtained by brushing the polymer solution onto the substrates. The films and the coatings were cured with moisture at room temperature and 80% RH. 

The seawater immersion test was conducted by putting the film or coating in a container filled with sterilized seawater, no organisms were present. The sterilized seawater preparation process was as follows. Unpolluted fresh seawater was taken from the Dalian Sea, then put in a dark room at room temperature for more than 3 months. It was then filtered and exposed to sterilization treatment at high temperature and high pressure. The salinity of seawater is 30 PSU. 

### 2.3. Characterizations

#### 2.3.1. Swelling Test

The PEG-PU film (the mass labeled m_0_) with a size of 20 mm × 20 mm × 2 mm was immersed in sterilized seawater. At regular intervals, the sample was taken out and rinsed with running water, then its surface water was soaked up with filter paper and weighed (the mass labeled m_t_).

The swelling rate of the film can be calculated by the following formula:

(1) $$\mathrm{Swelling}\;\mathrm{rate}\;\mathrm{in}\;\mathrm{mass}\;(S_{m})=\;\frac{m_{t}-m_{0}}{m_{0}}\times 100\%$$

The density of seawater was about 1 g/cm^3^ and the density of the PEG-PU film was about 1.08 g/cm^3^ which was measured in our laboratory. Thus, the swelling rate in volume $S_{v}\approx S_{m}$. The swelling degree is the swelling rate reached swelling equilibrium.

#### 2.3.2. X-ray Diffraction (XRD)

XRD patterns of the coatings were measured using a D/MAX Ultima X-ray diffractometer (RIGAKU Co., Ltd., Tokyo, Japan) equipped with CuKα radiation (λ = 1.54178 Å) as the X-ray source over a 2θ range of 5° to 90°. The scan speed was 0.15 s/step and the step width was 0.02°.

#### 2.3.3. Differential Scanning Calorimetry (DSC)

NETZSCH DSC 200 F3 thermal analyzer (NETZSCH Co., Ltd., Bavaria, Germany) was used to analyze the coating samples. Approximately 6–10 mg of sample was filled in aluminum pans and tested at a cooling/heating rate of 10 °C/min under dry nitrogen from −100 °C to 200 °C. DSC-NETZSCH_Proteus 4.8.2 analysis software was used to analyze and evaluate the glass transition temperature of the coatings from the DSC curves.

#### 2.3.4. Fourier Transform Infrared

Attenuated total internal reflectance Fourier transform infrared (ATR-FTIR) spectra of the samples were recorded using a Frontier PerkinElmer infrared spectrometer (PerkinElmer Co., Ltd., Waltham, MA, USA) with a resolution of 0.5 cm^−1^ and 128 scans. The ATR crystal was diamond with an end face angle of 45°. The air-facing side of the polymer film was placed against the crystal. In the FTIR analysis, integrated intensities of the absorbance bands were corrected for sample differences using the N–H and N–C mixing band near 1535 cm^−1^ as the normalizing factor.

#### 2.3.5. Atomic Force Microscope (AFM)

The surface morphology of the samples was observed by Nanoscope IIIa Scanning probe microscopy (Veeco Co., Ltd., New York, NY, USA), operating in tapping mode with software version 5.30r3sr3.

#### 2.3.6. Water Contact Angle

The measurements of the water contact angle were conducted using a JC2000 contact angle measurement system (Shanghai Zhongchen Digital Technic Apparatus Co., Ltd., Shanghai, China) at room temperature. A 3 μL droplet of distilled water/diiodomethane was placed on the surface of the coating sample. After stabilization, the image was recorded and the contact angle was measured by the sessile drop method. The surface energy was calculated by Owens two-liquid method.

#### 2.3.7. Tensile Test

The tensile properties were measured using Labthink XLM auto tensile tester (Labthink Co., Ltd., Jinan, China). The cast film specimens of 120 mm × 20 mm × 2 mm were tested at a speed of 50 mm/min, and their strain–stress curves were recorded. Moduli and strains at 100% of coatings were obtained from the curves.

#### 2.3.8. Marine Bacterial Test

Using fresh seawater as the bacterial solution, the antibacterial properties of the coatings were studied by measuring and comparing the OD600 of the bacterial solution attached on coatings. The coating samples were immersed in the fresh seawater for 24 h to cause the marine bacteria to adhere to the coating surface. Then, the samples were taken out and gently rinsed in sterilized deionized water to remove the unattached bacteria. Then, the samples were dried on a sterile clean bench, placed in the 2216E liquid medium tube and cultured in an incubator at 37 °C for 24 h so that the attached bacteria gradually moved into the liquid medium where they grew and multiplied. After that, the samples were taken out and the tube was shaken. The OD600 of the liquid medium containing the marine bacteria from the coating surface was then tested in order to estimate the antibacterial properties of the coatings.

## 3. Results and Discussion

### 3.1. Swelling in Seawater

**Figure 4:**
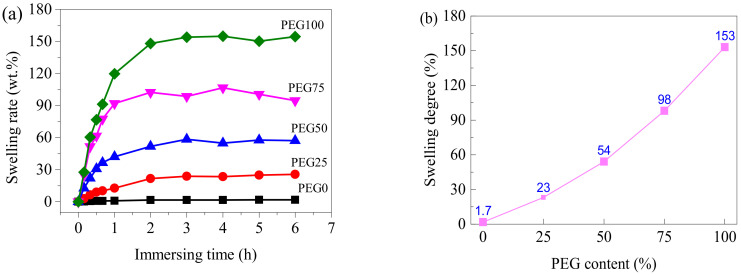
Swelling behavior of the PEG-PU films in seawater. (**a**) swelling rate with time; (**b**) swelling degree with PEG content.

**Figure 5:**
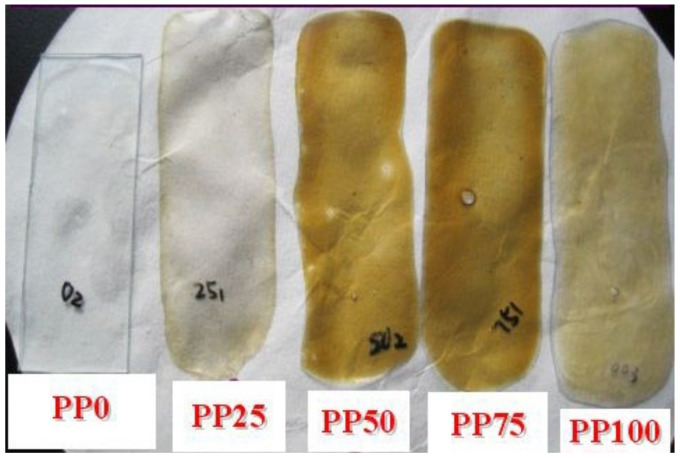
Appearance of the coatings immersed in seawater for 30 days.

The volume and mass of the PEG-PU films increased with PEG content and immersion time, after being immersed in seawater. Figure 4a shows the curves of the swelling rate of the PEG-PU films along with immersion time in seawater. It can be observed that the PEG-PU films rapidly swelled in seawater at an early stage. After about 2 h they reached swelling equilibrium, the swelling degrees of the samples with PEG contents of 0, 25, 50, 75 and 100% were 1.7, 23, 54, 98 and 153 wt.%, respectively (shown in Figure 4b). Note that the coatings PEG75 and PEG100 were peeled off from the glass slides after immersion in seawater for two hours due to the marked increase in volume. After 30 days, all the coatings containing PEG were peeled off the glass slides and became yellow, but the coating without PEG was still transparent and did not peel off (shown in Figure 5).

### 3.2. Microstructure

**Figure 6:**
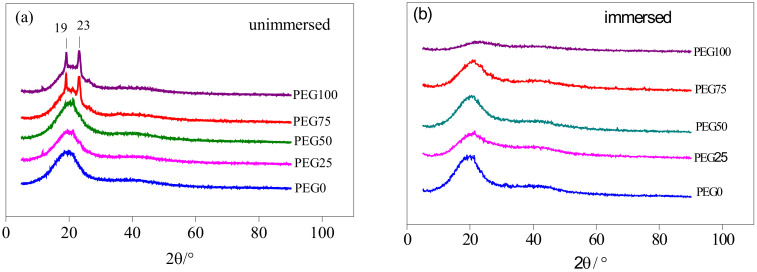
XRD patterns of coatings. (**a**) Not immersed; (**b**) Immersed in seawater for 30 days.

PEG is a polymer that crystallizes easily. It always shows the characteristic pair of Bragg reflections at 2θ = 19.3° and 23.5° in the X-ray diffraction patterns [29]. They belong to the (120) and (032) Miller planes of the PEG monoclinic unit cell. In the XRD patterns of the PEG-PU coatings shown in Figure 6a, there are a pair of peaks at 2θ = 19° and 23° for the PEG75 and PEG100 coatings, but no obvious peaks occur for the PEG0, PEG25 and PEG50 coatings. However, a broad amorphous diffuse scattering peak occurred (shown in Figure 6b) for each coating immersed for 30 days in seawater at around 2θ = 20°. It can be seen that some PEG chains stay close together and in a regular formation in the soft segments of the PEG-PU coatings in which PEG content is less than 50%, but the regular arrangements of the PEG chains in soft segments becomes disordered after immersion in seawater.

With increasing the PEG content, the crystallinity degree of the coatings increases (Table 1). The PEG structure is symmetrical and its chain segments are regular, so it has a good level of crystallinity. In contrast, the CH_3_ molecules of the PPG chain segments have a steric effect, which will hinder the regular arrangement of the chain segments, so the crystallinity is poor. After immersion in seawater, water molecules and ions in seawater provide a large number of hydrogen bond donors and acceptors, which makes NH, C=O, and C–O–C in polyurethane tend to form new hydrogen bonds with them, destroying the hydrogen bonds and crystals in the original polyurethane, which is not conducive to the directional arrangement of the molecules. Consequently, the crystallinity is greatly reduced. Thus, the degree of microphase separation and antifouling performance were affected.

**Figure 7:**
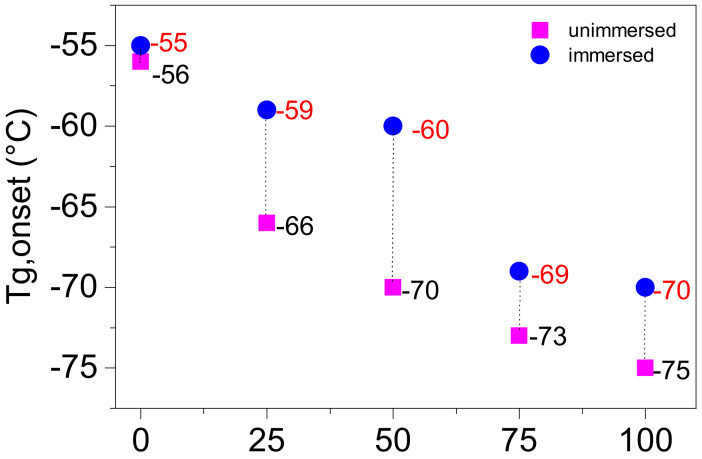
Glass transition temperature of the coatings.

The glass transition temperature of the coatings was obtained by DSC curves, as shown in Figure 7. The curves indicate that the glass transition temperature of the coatings decreased with increasing PEG content, and increased after immersion in seawater. The glass transition temperature at the stage of the lower temperature is mainly attributed to the irregular PPG and PEG soft segment domains. The glass transition temperature of PPG soft segment domains and irregular PEG soft segment domains corresponding to the coatings of PEG0 and PEG100 are, respectively, 56 and 75 °C. Thus, the irregular PEG soft segment domain of the coatings possesses a lower glass transition temperature than the PPG soft segment domains. Additionally, the glass transition temperature of the soft segment domains mixed PPG and irregular PEG for PEG25, PEG50 and PEG75 are 66, 70 and 73 °C, respectively, which are between the values for PEG0 and PEG100, and they also decrease with PEG content. The increases in the glass transition temperature of coatings after seawater immersion may be caused by the weakening of microphase separation that results in a greater level of mixing between the hard segment and the soft segment.

**Figure 8:**
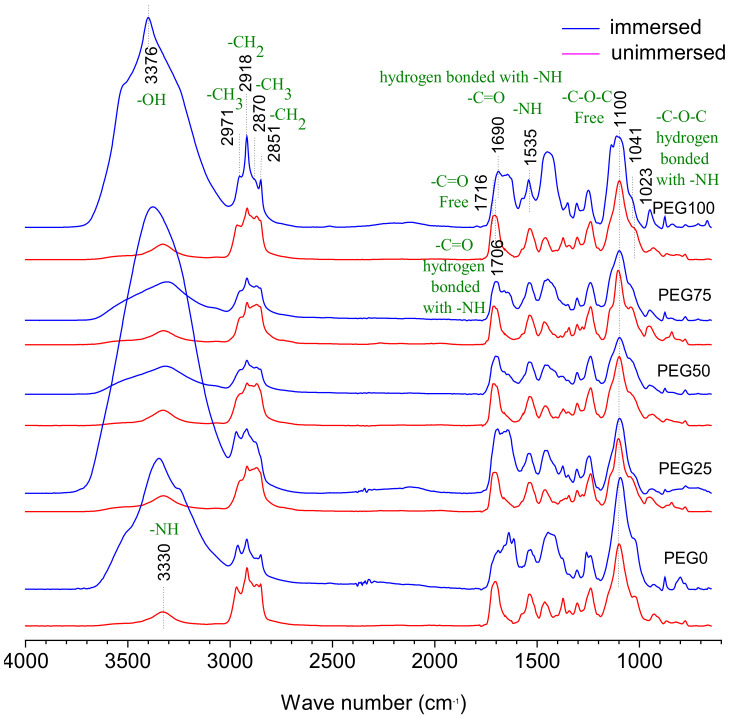
Attenuated total internal reflectance Fourier transform infrared (ATR-FTIR) spectra of the PEG-PU coatings.

The properties of polyurethane materials are closely related to the phase separation with nanoscale domains of the hard segment and soft segment, deriving from their incompatible composition [18]. Hydrogen bonding determines their phase separation. The high electronegativity of nitrogen atoms in the urethane or urea moiety withdraws NH bonded electrons and develops partial positive charge on the hydrogen, which thereby forms hydrogen bonding with the neighboring oxygen atoms. In all cases, the hydrogen atoms of the NH group in the urethane or urea linkages are the donor protons, while the acceptor group can be the carbonyl of urethane’s C=O, urea’s C=O or the oxygen atom of ether linkage when polyether is present as the soft segment. The hydrogen bonding interaction produces a physical crosslink, thereby reinforcing the polyurethane matrix and increasing the strength and stiffness [30]. It has been reported that the extent of hydrogen bonding of the urethane’s carbonyl and or urea’s carbonyl can approximate the degree of phase separation [31]. FTIR-ATR was used to probe the composition and determine the relative content of composition and the relative degree of phase separation of the material surface [32]. There are two types of hydrogen bonding in our PEG-PU coatings, one is the hydrogen bonding between the amino NH and the carbonyl C=O in the carbamic acid ester of the hard segment phase which can easily result in microphase separation owing to the formation of hard segment domains and soft segment domains. Another is hydrogen bonding between the amino NH in carbamic acid ester of hard segment phase and ether oxygen group of the soft segment phase, which goes against microphase separation. Figure 8 shows the FTIR spectra of the PEG-PU coatings. The red lines are the spectra of coatings cured at room temperature for more than 24 h. The blue lines are the spectra of coatings immersed in natural filtered seawater for 30 days and washed with deionized water, in which the samples of PEG50 and PEG75 were dried at room temperature for more than 24 h, and PEG0, PEG25, and PEG100 only had water sucked off their surface with filter paper. Distinct bands of asymmetric and symmetric methyl CH_3_ and methylene CH_2_ were observed at 2970 and 2870, 2851 and 2918 cm^−1^, respectively. The relative intensity of CH_2_ peaks at 2870 and 2918 cm^−1^ were enhanced with increasing PEG content. The band of hydrogen bonded NH was observed at about 3330 cm^−1^ and was obviously enlarged after immersion in seawater for PEG0, PEG75 and PEG100 because of the hydration layer around the hard segment. After seawater immersion, the free NH of the PEG25 and PEG50 coatings at the left shoulder of the peak at 3330 cm^−1^ were enhanced compared with those of the not-immersed ones, indicating that the degree of hydrogen bonding to NH was reduced due to immersion in seawater. The hydrogen bonded C=O with NH and free C=O were observed, respectively, at 1706 and 1716 cm^−1^. The band of hydrogen bonded C=O at 1716 cm^−1^ encompasses both disordered and ordered parts. With the increase in PEG content, a peak can be seen in the increase in free C=O relative to the hydrogen bonded C=O. After immersion in seawater, hydrogen bonded C=O peaks shifted to 1690 from 1706 cm^−1^ because the disordered hydrogen bonded C=O were broken by hydration. The band of free C–O–C and hydrogen bonded C–O–C with NH were observed, respectively, at 1100 and 1023 cm^−1^ and the peak intensity of the free C–O–C greatly exceeded the hydrogen bonded C–O–C. The bands do not seem to change significantly with increasing PEG content and seawater immersion, which suggests that the hydrogen bond between the C–O–C of soft segment and the NH of hard segment is very weak.

**Figure 9:**
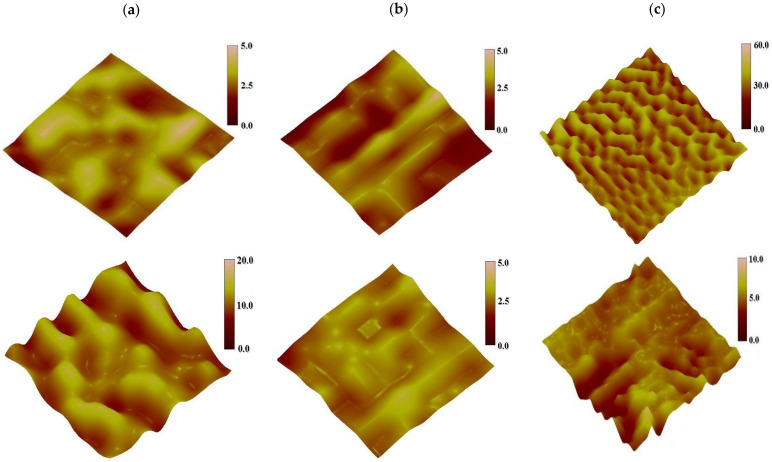
The surface height images of coatings not-immersed (up) and immersed (down) in seawater, (**a**) PEG0, (**b**) PEG50, (**c**) PEG100, tap mode, region of 1 μm × 1 μm.

The AFM height images of the coatings are shown in Figure 9, in which the bright convex surfaces are hard segment domains with high cohesive energy, the dark concave portion are soft segment domains. The cohesive force of the soft segment is smaller than that of the hard segment, so that the hard segment protrusion is uniformly dispersed in the soft portion on the segment base material. The microphase domains of the coatings have different characteristics in the three-dimensional topography of the AFM. For the PEG0 coating with no PEG, the irregular distribution of surface microphase domains are only distinguishable under the height of 5 nm, so it can be considered that there is no significant microphase separation, indicating that the soft segment and the hard segment are well mixed. As the PEG content increases, the distribution of surface microphase domains becomes regular, and is distinguishable under the height of 5 nm for PEG50 and 60 nm for PEG100. The width of the microphase domains was reduced to about 50 nm for PEG100, from about 110 nm for PEG0 and PEG50, showing that the crystalline PEG promotes the microphase separation of coatings. After seawater immersion, the distribution of surface microphase domains became more regular for PEG0 and PEG50 and disordered for PEG100, and the distribution is distinguishable under the height of 20 nm for PEG0, under the height of 5 nm for PEG50, and under the height of 20 nm for PEG100. Obviously, seawater immersion can help to improve the microphase separation of coatings without PEG, however, this goes against the microphase separation of coatings with crystalline PEG.

### 3.3. Surface Hydrophily

**Figure 10:**
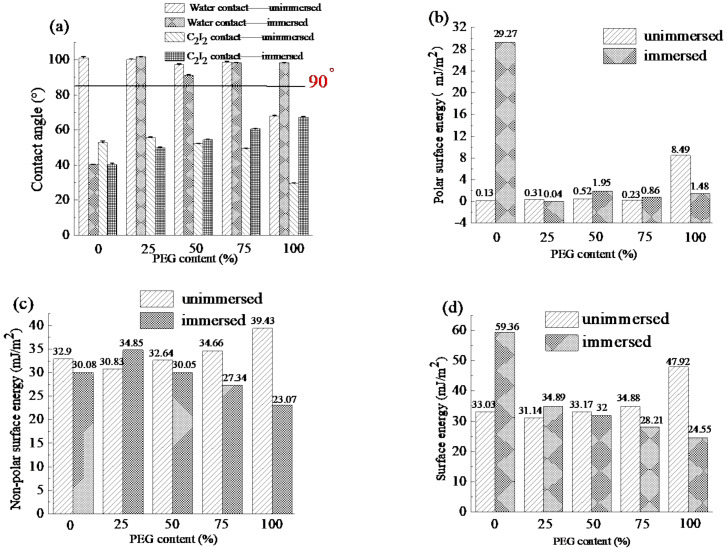
The contact angle and surface energy of coatings not immersed and immersed in seawater, (**a**) Contact angle, (**b**) Polar surface energy, (**c**) Non-polar surface energy, (**d**) Surface energy.

The contact angle and surface energy of the coatings are shown in Figure 10. Before immersion, with the increase in PEG content, the water contact angle decreased due to the hydrophilicity of PEG, the corresponding surface energy also increased. After immersion, the water contact angle of the PEG0 coating without PEG decreased significantly because the roughness increased. However, the coatings with PEG can maintain the high water contact angle because the immersion promoted the mixing of hard and soft segments. With the increase in PEG content, the surface energy also decreased from 59.36 to 24.55 mJ/m^2^. According to the Baier curve [33], the antifouling effect was optimal when the surface energy was between 23 and 25 mJ/m^2^. When the surface energy is over 25 mJ/m^2^, with the increase in the surface energy, the antifouling ability gradually decreases.

**Figure 11:**
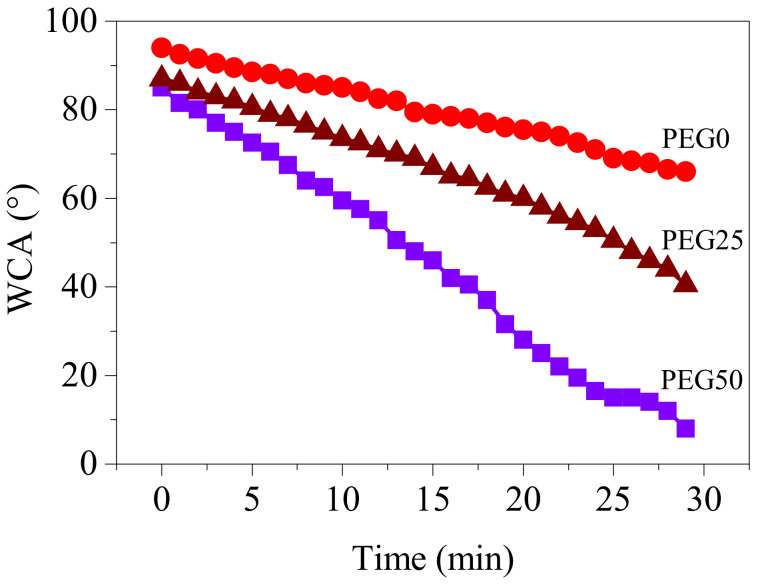
Variation of water contact angle with time for coatings.

The surface hydrophily of the coatings was characterized by variation of the water contact angle with time, as shown in Figure 11. The water contact angle of the coatings decreased with time, and the higher PEG content in coatings, the faster the water contact angle decreased. The beginning water contact angles for PEG0, PEG25, and PEG50 were 94°, 87°, and 85°, respectively. As the water droplets spread over time, the contact angle decreased gradually due to the migration of the PEG segment to the interface between water and coating, after 30 minutes, the water contact angle of PEG0, PEG25 and PEG50 decreased to 66°, 41° and 8°, respectively, indicating that when the PEG content reaches 50%, the coating has excellent hydrophily.

### 3.4. Mechanical Property

**Figure 12:**
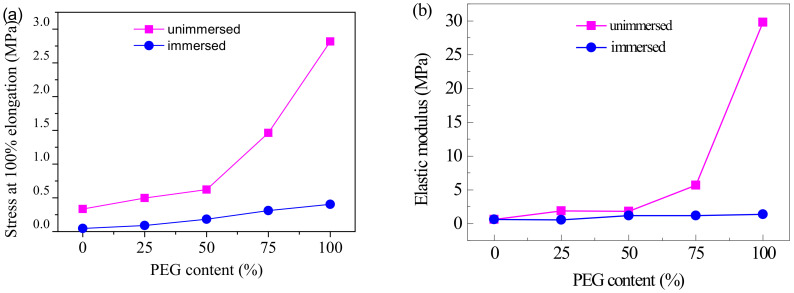
Tensile stress at 100% (**a**) and elastic modulus (**b**) of coatings with PEG content.

Tensile stress at 100% and elastic modulus of coatings with PEG content from the stress–strain curves are shown in Figure 12. The stress and modulus of coatings both increased with PEG content, and that increased even faster when the PEG content is over 50%. The stress values of PEG50 and PEG100 were, respectively, 1.9 and 8.5 times bigger than that of PEG0. The modulus of PEG50 and PEG100 were, respectively, 3.2 and 52.3 times bigger than that of PEG0. After immersion in seawater, the stress and modulus of the coatings all lowered, and increased with PEG content at a relatively slower rate. The stress of PEG0, PEG25, PEG50, PEG75 and PEG100 were, respectively, reduced from 0.33, 0.50, 0.62, 1.46 and 2.81 MPa to 0.05, 0.09, 0.18, 0.31 and 0.40 MPa, and the modulus of PEG0, PEG25, PEG50, PEG75 and PEG100 were, respectively, reduced from 0.57, 1.88, 1.83, 5.67 and 29.78 MPa to 0.57, 0.56, 1.19, 1.16 and 1.38 MPa. It can be seen that the strength and rigidity of the coatings were significantly improved by the crystallized PEG in the soft segment, while the non-crystalline PEG—after immersion in seawater—made the coatings softer and more elastic. Because the crystallinity of PEG was higher than that of PPG, most of the molecular chains were arranged in an orderly and tight fashion. The strong intermolecular interactions led to difficulty in chain movement. Thus, the elastic modulus and stress at 100% elongation were increased. PEG chains in the soft segments became disordered after immersion in seawater. The crystallinity was greatly weakened, so the elastic modulus and stress at 100% elongation were also reduced.

### 3.5. Antibacterial Performance

**Figure 13:**
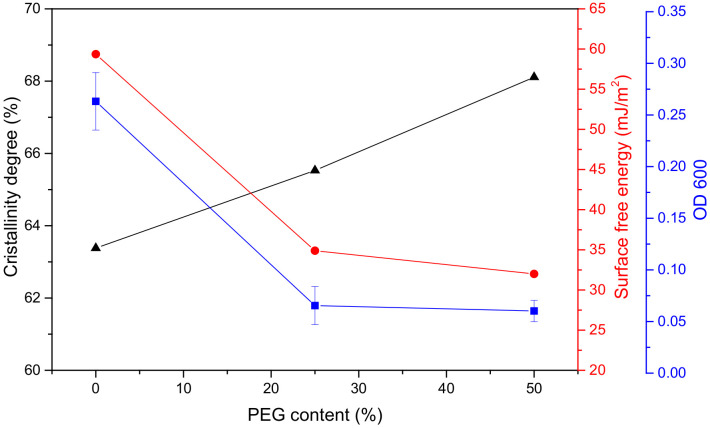
Bacteria attached (OD600) to the coatings, crystallinity and surface free energy with PEG content.

**Figure 14:**
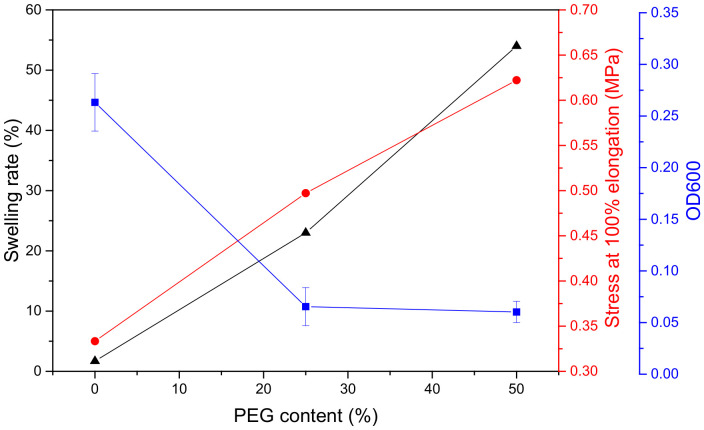
Bacteria attached (OD600) to the coatings, swelling rate and stress at 100% elongation with PEG content.

The OD600 values of the liquid medium containing marine bacteria from the coating surface are shown in Figure 13 and Figure 14. The OD600 values of PEG25 and PEG50 were significantly lower than that of PEG0 without PEG; down 75 and 77%, respectively. Similarly, the surface energy decreased. After adding PEG, the crystallinity of the coatings and the degree of microphase separation increased, the swelling rate and stress at 100% elongation also increased, and the coating became rougher, which was not conducive to fouling biological attachment. However, the PEG content increased from 25 to 50%, and the OD600 value decreased by only 2%. Obviously, the surface energy decisively affected bacterial attachment on the coating. To sum up, a certain amount of PEG incorporated into the soft segment can obviously improve the antibacterial property of the coating, but adding more PEG has little significance.

### 3.6. Mechanism of Interaction between the Coating and Seawater

In PEG-PU coatings, the amorphous PEG chains facilitated the formation of hydrogen bonding between the amino NH in the carbamic acid ester of the hard segment phase and the ether oxygen group of the soft segment phase, which goes against the microphase separation. However, the crystallized PEG chains facilitated the formation of hydrogen bonding between the amino NH and the carbonyl C=O in carbamic acid ester of the hard segment phase which resulted in easy microphase separation. Thus, PEG-PU coatings were constituted of hard segment domains with hydrogen bonding, a crystallized PEG phase in soft segments, and a mixing phase with hydrogen bonding between hard segments and amorphous PEG and PPG of soft segments (as shown in Figure 6, Figure 7, Figure 8 and Figure 9). Due to the excellent crystallization performance of PEG, with the increase in PEG content, the crystallinity, microphase separation degree, roughness of the coatings increased. Therefore, they had better antifouling performance.

**Figure 15:**
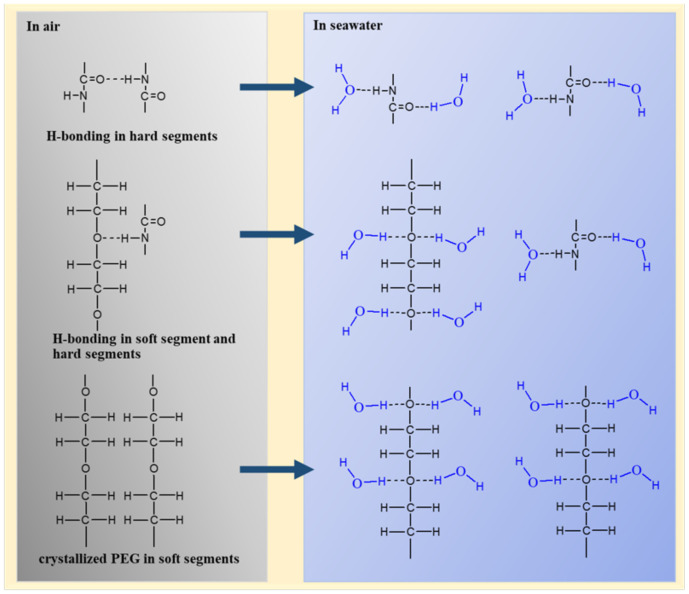
Schematic mechanism of interaction between coating and seawater.

When the coatings were immersed in seawater, large quantities of water molecules went into the coating from water sorption of PEG, then formed hydrogen bonds with the amino NH and the carbonyl C=O in the carbamic acid ester of the hard segment, and the ether oxygen group of the soft segment phase, as shown in Figure 15, which destroyed the original hydrogen bonding and crystallization and formed uniformly free mixing microstructures. At the initial stage of immersion, the coating relied on the microphase separation for antifouling. With the increase in immersion time, the degree of microphase separation weakened and the antifouling performance decreased. Due to the strong water absorption of PEG, the coating gradually absorbed water to reach the swelling equilibrium, forming a hydration layer for antifouling.

## 4. Conclusions

In poly(ethylene glycol)-segmented polyurethane (PEG-PU) coatings, PEG and PPG mixed in a certain proportion acted as the soft segment, which can ensure the basic performance and has good antifouling performance. With the increase in PEG content, the stress at 100% and elasticity modulus and swelling degree in seawater increased by a maximum of 8.5 times, 52.3 times and 153%, respectively. In particular, the water contact angle decreased to 8°, showing that the coating has excellent hydrophily, so it helped to form a hydration layer and avoided protein adsorption. PEG had a good crystallinity. After adding PEG, the crystallinity, microphase separation and roughness of the coatings increased, forming a dynamic equilibrium hydrophilic rough surface. At the same time, the coatings had a low surface energy, these advantages can effectively improve the antifouling performance. After immersion in seawater, with the increase in PEG content, the coatings became softer and more elastic, the surface energy was gradually reduced, and the swelling degree gradually increased. These changes were conducive to the formation of the hydration layer, enhancing the antifouling performance. However, excessive swelling caused it to fall off the substrate and lose its application value. At the same time, the hydrophilicity of PEG made water molecules enter the coating and form hydrogen bonding with the amino NH and the carbonyl C=O in the carbamic acid ester of the hard segment and the ether oxygen group of the soft segments so that the original hydrogen bonding and crystallization were destroyed. With the increase in PEG, the degree of microphase separation and roughness of the coatings decreased gradually, which resulted in reduced antifouling performance. After antibacterial performance, the coating with 25% of PEG incorporated in the soft segment markedly improved the antibacterial properties of the coating, but adding more PEG has little significance.

## Figures and Tables

**Table 1:** The crystallinity of samples before and after seawater immersion.

Crystallinity Degree (%)	PEG0	PEG25	PEG50	PEG75	PEG100
Unimmersed	63.38	65.53	68.11	73.07	79.19
Immersed	5.65	5.66	5.25	8.03	2.09

## Data Availability

Data is contained within the article.
